# Role of Transcutaneous Electric Nerve Stimulation in Pain and Pulmonary Function in Patients after Bariatric Surgery

**DOI:** 10.1155/2022/9138602

**Published:** 2022-06-02

**Authors:** Cesar Antonio Luchesa, Agnaldo José Lopes

**Affiliations:** ^1^Rehabilitation Sciences Post-Graduation Programme, Augusto Motta University Center (UNISUAM), Rio de Janeiro, Brazil; ^2^Rehabilitation Center, University Center Fundação Assis Gurgacz (FAG), Cascavel, Brazil; ^3^Medical Sciences Post-Graduation Programme, School of Medical Sciences, State University of Rio de Janeiro (UERJ), Rio de Janeiro, Brazil

## Abstract

**Background:**

Changes in lifestyle, a carbohydrate-rich diet, and decreased physical activity are part of the context that led to an obesity pandemic. Treating obesity is a task that requires multidisciplinary care; however, in many cases, conventional therapy has no effect, requiring surgical intervention. This, in turn, is not without risks and causes important changes in lung function. Therefore, the aim of this study is to evaluate the effect of electroanalgesia through conventional transcutaneous electrical nerve stimulation (TENS) on the pain and lung function in the postoperative period of bariatric surgery.

**Methods:**

This is a controlled and blinded clinical trial with 66 subjects who underwent bariatric surgery. The participants were randomized into 2 groups as follows: an intervention group treated with TENS (*n* = 33) and a placebo group (*n* = 33). The participants underwent 4 TENS sessions, and the effect on pain was assessed using a visual analogue scale (VAS pain). Lung function was measured by spirometry.

**Results:**

There were no differences between the 2 groups regarding time of surgery and time of mechanical ventilation. Compared to placebo, TENS reduced pain in the intervention group (*p* = 0.001). Regarding the effect of electroanalgesia on pulmonary function, the spirometric parameters were similar between the groups. However, regarding muscle strength between the preoperative and postoperative periods, maximal inspiratory pressure (MIP) was maintained in the intervention group and decreased in the placebo group (*p* = 0.03). Compared with that in the intervention group, the respiratory rate in the placebo group increased during the application of TENS (*p* = 0.003).

**Conclusion:**

Electroanalgesia reduces pain in patients who underwent bariatric surgery. Importantly, in these patients, the MIP is maintained between the preoperative and postoperative periods. However, electroanalgesia does not contribute to improvements in spirometric data. This trial is registered with NCT04800640.

## 1. Introduction

Obesity is one of the main public health problems worldwide, with a multifactorial aetiology resulting from changes in eating habits, social factors, psychological changes, and epigenetic changes [1]. It has well-established complications, including an increased risk of hypertension, dyslipidaemia, and diabetes mellitus (metabolic syndrome), coronary artery disease, gallbladder disease, degenerative joint injuries, and obstructive sleep apnoea, in addition to socioeconomic and psychosocial impacts [2].

When undergoing upper abdominal surgery, obese individuals are more susceptible to pulmonary repercussions caused by anaesthesia and by the surgical procedure itself. In obese individuals, anaesthesia accentuates the reduction in functional residual capacity (FRC), promoting the closure of small airways. This leads to a higher degree of hypoxemia than that observed in nonobese patients, in addition to a higher incidence of atelectasis [3]. In the postoperative period of Fobi Capella gastroplasty, obesity has important consequences on lung function, with decreased lung volume and capacity and reduced respiratory muscle strength [4].

Pain is a factor with a strong negative impact on the postoperative evolution of patients after abdominal surgery. In particular, pain occurs in patients after surgery involving the upper abdomen, even when using analgesic medication [5]. Transcutaneous electrical nerve stimulation (TENS) is a low-cost physiotherapeutic resource with few contraindications and is widely used for pain relief [6]. In the postoperative period, the effect of TENS is by acting on nociceptive pain. Thus, TENS can reduce pain through some mechanisms including modification of sympathetic activity by reducing the activation of local *α*2A-noradrenergic receptors, decrease in excitatory neurotransmitter (substance P), and activation of peripheral inhibitory *μ*-opioid receptors [7].

There seems to be a relationship between pain and lung volume and capacity in the postoperative period of some types of surgery. After the use of TENS, improvements in pain and increases in tidal volume (VT) have been reported in the postoperative period of cardiopulmonary [8, 9]. The efficacy of TENS, with the benefit of electrostimulation, has also been demonstrated in thoracic surgery, especially in reducing the need for opioids and increasing the effectiveness of cough exercises during physical therapy [10]. The use of electroanalgesia has also been shown to reduce pain, especially cough-related pain [11, 12]. Because it has few contraindications, is noninvasive, and is easy to apply, TENS could be an important tool in the relief of surgical pain, especially pain associated with surgeries of the upper abdomen. Furthermore, the use of TENS appears to exert a reduction in blood levels of proinflammatory cytokines causing a protective factor in relation to inflammation [13], which is a condition closely related to obesity. Given the benefits of TENS in pain relief, we hypothesized that TENS would result in the maintenance of thoracic mobility, reducing the undesirable effects of surgery on lung function. Thus, the aim of our study was to evaluate the effect of TENS on the pain and lung function of open bariatric surgery patients in the postoperative period.

## 2. Materials and Methods

### 2.1. Study Design and Participants

This was a blinded randomized controlled clinical trial conducted from March 2021 to August 2021 at São Lucas Hospital Foundation, Cascavel, Brazil. Individuals in the postoperative period of bariatric surgery who were admitted to the intensive care unit (ICU) and ≥18 years of age were included. The exclusion criteria were as follows: age > 65 years; haemodynamic instability in the postoperative period; use of mechanical ventilation for more than 24 hours; contraindications to the use of TENS (presence of a cardiac pacemaker and hypersensitivity to the procedure); already undergone electroanalgesia; previous history of epilepsy; pregnant women during the first trimester; and Glasgow coma scale score < 15 points.

This study was approved by the Research Ethics Committee of the Assis Gurgacz University Center, approved under number CAAE-11613219.0.0000.5219. All participants freely signed an informed consent form in accordance with regulation 466/12 of the National Health Council and Helsinki Declaration, after being informed of procedures and objectives of the study. This study is registered at http://www.clinicaltrials.gov (NCT04800640).

### 2.2. Randomization

The sample was randomized into 2 groups: (1) intervention group, composed of patients who received electroanalgesia via TENS, and (2) placebo group, composed of individuals who received placebo TENS.

Randomization was performed by an independent research assistant who did not participate in any other section of this study. An allocation concealment protocol with sequentially numbered, opaque, sealed envelopes and permuted blocks of 4 was used to randomize participants to groups. Enrolled participants were only informed that they would receive one of the two types of application; consequently, they were not aware of their treatment allocation. The examination was done by an independent physical therapist before and after the protocol of 4 sessions; this examiner did not follow the application and did not know which group the patients had been included in. Participants, clinical outcome evaluators (vital signs, visual analogue scale for pain-VAS pain, manovacuometry, and spirometry), and statistics were blinded to randomization, since it was not feasible to blind the physical therapists who administered TENS.

### 2.3. Measurements

The participants underwent a physical therapy assessment that consisted of acquiring anthropometric and demographic data and intraoperative data and monitoring vital signs. TENS or placebo was applied only if the participant had been in ICU for longer than 12 hours. After this period, protective measures, including an evaluation of vital signs (blood pressure, partial oxygen saturation, and heart rate) and an assessment of the level of consciousness (Glasgow scale), were implemented. The level of pain was also assessed using a VAS pain before and after each application of electroanalgesia and placebo. Respiratory muscle strength was measured by manovacuometry and spirometry in the preoperative period and on the second postoperative day after the last application of electroanalgesia.

Spirometry was assessed using a portable device (Micromedical, Microloop model, Brasília, Brazil). All tests were conducted in accordance with the standards of the American Thoracic Society [14], and national reference equations were adopted [15]. Respiratory muscle strength was assessed using a digital manometer (Globalmed, model MVD 300, Porto Alegre, Brazil), with the patient seated and using a nose clip. To interpret the maximum inspiratory pressure (MIP) and maximum expiratory pressure (MEP) values, national reference equations were adopted [16].

### 2.4. Interventions

After the physical therapy evaluation, patients in the intervention group received 4 sessions of active TENS, and patients in the other group received 4 sessions of placebo TENS (KLD Endophasys model NMS.0501, Jardim Camanducaia, São Paulo, Brazil). The TENS protocol was implemented 1 hour after the application of drug analgesia. The 4 electroanalgesia and placebo sessions occurred at 10 am in the morning and at 4 pm in the afternoon for 2 consecutive days. Four Asktus electrodes (elastic hydrogel adhesive; 5 × 5 cm) were positioned 3 centimetres from the surgical incision based on the recommendations by Ferreira and Beleza [17]. Electroanalgesia was performed with the participants seated in an armchair and only after their vital signs had been stable for 10 minutes. For the application of TENS to relieve acute pain, a conventional current with a frequency equal to 80 Hz and pulse width equal to 200 *μ*s was used [18]. Once patients showed sensory accommodation, however, they were encouraged to increase the intensity to a strong but comfortable intensity, just below the pain threshold, and continue to increase the intensity as tolerated [19]. Still in relation to the perceptual-sensory adaptation to TENS, the intensity was titrated upwards during therapy in order to obtain a greater analgesic effect [20]. An asymmetric bipolar pulse was applied for 30 minutes, and the intensity was maintained at a strong sensory level; after 10 minutes of application, a continuous tingling sensation is felt in the abdominal region [21]. All participants followed the analgesic medication regimen.

For individuals in the placebo group, the same procedures and positioning of the electrodes were adopted, differing only in the current intensity. The device remained on for 30 minutes of therapy, but no intensity was applied, so as to generate a placebo effect.

After the end of the 4 electroanalgesia and placebo sessions, the participants were reevaluated (VAS pain, manovacuometry and spirometry) to assess the effect of TENS on pain, MIP, MEP, and spirometric parameters.

### 2.5. Data Analysis

Data normality was assessed using the Shapiro-Wilk test. The results are expressed as the mean ± standard deviation or median (interquartile ranges) based on the Gaussian or non-Gaussian distribution of each variable. Comparisons between groups by age, height, weight, and body mass index (BMI) were conducted using Student's *t*-test for independent samples, the Mann–Whitney test for numerical data, and Fisher's exact test for categorical data. Pulmonary function, pain, and haemodynamic data were evaluated by the Wilcoxon signed-rank test, and comparisons of the absolute deltas (post-TENS minus pre-TENS measurements) between the groups were conducted using the Mann–Whitney test. The significance level adopted was 5%. Statistical analyses were performed using SAS 6.11 (SAS Institute, Inc., Cary, North Carolina, USA).

## 3. Results

Among the 78 patients eligible for the study, 11 were excluded because they were discharged before the completion of the protocol, and 1 patient was discharged due to postoperative complications. Thus, 66 patients were included (50 women and 16 men) and randomized into 2 groups: (1) intervention group, composed of 33 participants (7 men and 26 women), and (2) placebo group, composed of 33 participants (9 men and 24 women).

The demographic and anthropometric data of the participants in the preoperative period and in the intraoperative period (time of surgery and time of mechanical ventilation) are provided in Table 1. There were no differences between the 2 groups regarding height, weight, BMI, time of surgery, and time of mechanical ventilation. There was a statistically significant difference between groups in relation to age, with a slightly higher mean age for participants in the placebo group (36.2 ± 8.5) than in the TENS group (41.3 ± 11.2, *p* = 0.040). The pulmonary function, pain, and haemodynamic data for the 2 groups before the application of electroanalgesia and placebo were similar (Tables 2 and 3).

**Figure 1:**
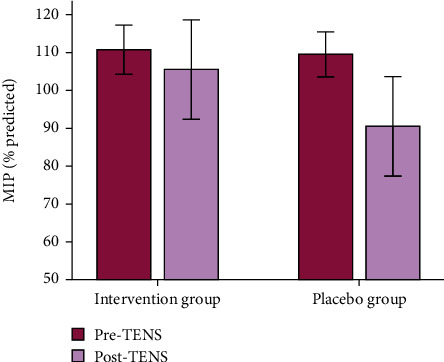
Comparisons in maximum inspiratory pressure (MIP) measurements before (pre-TENS) and after (post-TENS) electroanalgesia sessions (absolute delta) between the intervention and placebo group (*p* = 0.03).

Regarding the effect of electroanalgesia on pulmonary function, lung volume and capacity were similar between the groups. However, regarding muscle strength in the preoperative and postoperative periods, the intervention group maintained the MIP levels within the normal range after TENS [absolute delta of 0 (-29–5) vs. -5.5 (-46–0), *p* = 0.03] (Figure 1). The data for pulmonary function and muscle strength before and after TENS or placebo are provided in Table 2.

**Figure 2:**
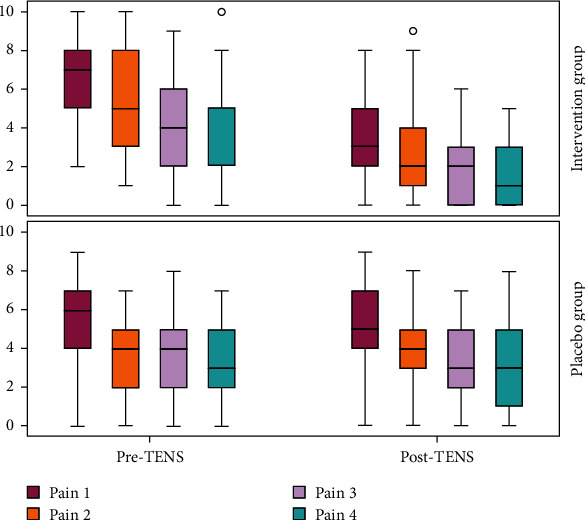
Comparisons between pain scale evaluations (VAS) before (pre-TENS) and after (post-TENS) 4 electroanalgesia sessions in the intervention group and in the placebo group (*p* < 0.001).

Compared to placebo, TENS reduced pain after all sessions, as verified using the visual analogue scale (*p* < 0.001) (Figure 2). Additionally, in the placebo group, the respiratory rate increased after the third session.  Table 3 provides the pain scale and haemodynamic data before and after TENS.

To provide context for interpreting the null findings, a post hoc power analysis was performed using GPower 3.1.1 software. Considering a type-I error of 5% and type-II error of 10% (study power of 90%), a minimum sample of 46 participants (23 per group) is necessary to observe a mean difference of at least 2 (SD of 2) in pain intensity between groups after the 4-session intervention.

## 4. Discussion

The main findings of the present study were that the use of electroanalgesia reduced pain levels in patients. Compared with placebo, TENS did not result in improvements in spirometric parameters. However, there was a positive effect of TENS on the maintenance of inspiratory muscle strength. In addition, the nonapplication of TENS (i.e., placebo) resulted in an increase in the respiratory rate. To our knowledge, this is the first study that investigates the use of TENS and evaluates its effect on pain and pulmonary function and haemodynamic parameters in patients in the postoperative period of open bariatric surgery.

In addition to obesity-related pulmonary functional changes, bariatric surgery amplifies restrictive ventilatory damage, either by tissue injury caused by the surgical incision or by the effects of anaesthesia, operative time, mechanical ventilation time, or pain. To relieve postoperative pain, one physiotherapeutic resource is the use of electrical currents to reduce pain stimuli and produce a morphine-like effect. Borges et al. evaluated the effect of TENS in patients in the postoperative period of abdominal surgery and found a reduction in postoperative pain in patients who received active TENS compared with those who received placebo and those in the control [22]. In our study, electroanalgesia reduced the pain levels in patients after all sessions; this result was not observed in the placebo group. The “theory of gates,” postulated by Melzack and Wall in 1965, was one of the first attempts to explain the effect of TENS [23]. Currently, there is a lot of knowledge about the peripheral, segmental, and central mechanisms regarding the use of TENS. It activates multiple central inhibitory pathways in spinal cord, rostral ventral medulla, and cortical sites and reduces central sensitization measured in nociceptive dorsal horn neurons simultaneously to reduce pain and hyperalgesia [24, 25]. The responses of the use of TENS in the activation of the spinal cord would result in indirect inhibition of the production of substance P [26]. Thus, the release of serotonin, norepinephrine, and gamma-aminobutyric acid (GABA) occurs in the dorsal horn neurons of the spinal cord. Activation of GABA_A_ receptors could be related to decreased production of glutamate and other excitatory amino acids, resulting in decreased pain; this demonstrates that the application of TENS to the patient's skin results not only in local and spinal cord but also supraspinal action [7, 24, 27].TENS also modulates nociceptive input at peripheral sites by peripheral impulse blockade and at segmental sites by “spinal gating” [27]. TENS includes the transmission of electrical current through the skin acting on peripheral mechanoceptors conducted by the A-*β* fiber to a set of interneurons; these, in turn, inhibit retransmission at the medullary level of painful stimuli conducted by A-delta and type C fibers [7, 24, 27].

The physiological effects of electrotherapy on the neurological and musculoskeletal systems are capable of reducing pain, which explains, at least in part, our results. Notably, other positive effects of TENS in patients in the postoperative period include muscle stimulation, vasodilation, reductions in oedema, reductions in reflex inhibition, facilitation of soft tissue injury healing, and facilitation of fracture consolidation [23].

Similar to our study, other authors have also evaluated the effect of TENS in the postoperative period of different surgeries. Tokuda et al. evaluated 48 patients in the postoperative period of abdominal surgery, comparing an intervention group (TENS) with a placebo group [28]. These authors assessed postoperative pain and conducted lung spirometry; TENS significantly reduced pain and increased the forced vital capacity (FVC) and VT. Luchesa et al. evaluated the effect of TENS on pain and pulmonary function in 30 patients (intervention group and placebo group) in the postoperative period of coronary artery bypass surgery [29]. They observed a reduction in pain levels in the intervention group; however, there were no improvements in the spirometric parameters of these patients. Similarly, in this study, we did not observe any significant effect on lung volume and capacity measured by spirometry, although there was a less pronounced reduction in FVC in the intervention group than in the placebo group. Regarding pain improvement, results similar to those in this study were reported by Tonella et al., who randomized their patients into 3 groups (intervention, control, and contrast/placebo) and found a reduction in pain in the postoperative period of abdominal surgeries and a reduction in pain when patients coughed [5]. Therefore, it seems reasonable to offer electroanalgesia as an adjunct to standard treatment for postoperative pain, mainly because it is a low-cost procedure and has a favorable safety profile compared to long-term medication [6]. A meta-analysis showed that electroanalgesia administered at strong and subnoxic intensity with adequate frequency can significantly reduce analgesic consumption for surgical pain [30].

The effects of pain result in a worsening of thoracic dynamics and consequently a reduction in the mobility of respiratory muscles, generating a reduction in inspiratory and expiratory pressures [2]. This has been well documented in gastroplasty surgery, with respiratory muscle strength reducing by approximately 50% on the first postoperative day and by approximately 25% on the second postoperative day [4]. Interestingly, in this study, the MIP values in the intervention group in the postoperative period were similar to those in the preoperative period, a finding that can be attributed at least in part to the effect of TENS because this finding was not observed in the placebo group. Similar to our results, Cipriano et al. demonstrated that TENS effectively controlled pain in the postoperative period of cardiac surgery and maintained muscle strength [12].

With a reduction in pain, patients should exhibit improvements in haemodynamic parameters, including respiratory rate, blood pressure, and heart rate. In our study, the respiratory rate in patients in the placebo group increased, suggesting a positive haemodynamic effect of TENS, i.e., maintaining the respiratory rate at a normal level. As shown by Fiorelli et al., significant incisional pain reduces chest mobility, causing depressed respiratory function, thus resulting in an inability to breathe deeply and cough effectively [31]. This situation can lead to significant alveolar collapse, severe hypoxemia, and severe postoperative pulmonary complications. One possible explanation for the positive effects of electroanalgesia is that TENS directly impacts rib cage mechanics and, consequently, ventilatory patterns. Notably, to our knowledge, no previous study has evaluated in detail the effects of TENS on haemodynamic parameters in the postoperative period.

As with any study, ours also has its limitations. First, the sample was relatively small, and the study included only 1 centre, which makes it difficult to generalize our results. Second, although the 2 groups were matched for anthropometric measurements, the group that received TENS had a mean age slightly lower than that of the placebo group. Despite these limitations, this study serves as a starting point for other randomized controlled trials with a greater number of patients undergoing other types of weight reduction surgery, such as laparoscopic surgery, vertical gastrectomy, and duodenal switch.

## 5. Conclusions

Our results indicate that the use of electroanalgesia reduces pain levels in patients. Although there was no change in spirometric parameters, there was a protective effect of TENS on respiratory muscle strength, with the maintenance of MIP values within the normal range. In addition, there may be an effect of TENS on haemodynamics because of its nonuse (placebo) affected tachypnoea. Based on the results of this study, electroanalgesia is a valuable tool in pain relief and the maintenance of respiratory muscle strength in the postoperative period of open bariatric surgery.

## Figures and Tables

**Table 1:** Demographic and anthropometric data, pulmonary function data, pain scale scores, and haemodynamic data in the preoperative period and variables measured in the intraoperative period.

	Intervention group (*n* = 33)	Placebo group (*n* = 33)	*p* value
*Demographic and anthropometric data*			
Male/female	7/26	9/24	0.89
Age (years)	36.2 ± 8.5	41.3 ± 11.2	**0.040**
Height (m)	1.63 ± 0.09	1.62 ± 0.08	0.39
Weight (kg)	116.9 ± 22.1	113.1 ± 19.2	0.45
BMI (kg/m^2^)	43.5 ± 4.9	43.3 (±6.4)	0.87
*Intraoperative data*			
ST (min)	135 (117–155)	120 (120–146)	0.79
MVT (min)	145 (125–175)	144 (120–161)	0.68

The results are expressed as the means ± SD or median (interquartile range); BMI: body mass index; ST: surgery time; MVT: mechanical ventilation time.

**Table 2:** Lung volume and capacity and muscle strength before and after the application of TENS and placebo.

	Intervention group (*n* = 33)			Placebo group (*n* = 33)				
	Pre-TENS	Post-TENS	Absolute delta	Pre-TENS	Post-TENS	Absolute delta	*p* value^∗^	*p* value^#^
*Spirometry*								
FVC (% predicted)	95 (88–105)	56 (39–69)	-39.0 (-53–-30)	96 (87–104)	52 (42–62)	-40.5 (-55–-26)	0.38	0.80
FEV_1_ (% predicted)	98 (89–111)	54 (44–68)	-43.0 (-54–-29)	101 (91–109)	54 (44–70)	-46 (-54–-21)	0.61	0.82
FEV_1_/FVC (%)	105 (100–107)	109 (96–115)	3.7 (-2–10)	105 (101–110)	113 (106–118)	7.1 (-1–16)	0.35	0.25
PEF (% predicted)	108 (89–125)	41 (30–58)	-63.0 (-83–-39)	103 (91–115)	40 (26–54)	-57.5 (-78–-39)	0.66	0.69
*Respiratory muscle strength*								
MIP (% predicted)	120 (110–120)	120 (72–123)	0 (-29–5)	120 (100-120)	113 (42–120)	-5.5 (-46–0)	0.79	**0.03**
MEP (% predicted)	120 (90–120)	77 (50–103)	-22.0 (-55–-6)	100 (85-120)	73 (56–96)	-27.5 (-50–0)	0.51	0.82

The results are expressed as the median (interquartile range); FVC: forced vital capacity; FEV_1_: forced expiratory volume in 1 second; PEF: peak expiratory flow; MIP: maximum inspiratory pressure; MEP: maximum expiratory pressure. ^∗^Comparison between the 2 groups in relation to pre-TENS measurements. ^#^Comparison between the 2 groups in relation to absolute delta values (post-TENS minus pre-TENS measurements).

**Table 3:** Pain scale scores and haemodynamic data before and after the application of TENS and placebo.

	Intervention group (*n* = 33)			Placebo group (*n* = 33)				
	Pre-TENS	Post-TENS	Absolute delta	Pre-TENS	Post-TENS	Absolute delta	*p* value^∗^	*p* value^#^
*Pain scale assessment*								
Pain 1 (score)	7 (4.5–8)	3 (2–5.5)	-2 (-3–-1.5)	6 (3.8–7)	5 (3.8–7)	0 (0–0)	0.067	**<0.001**
Pain 2 (score)	5 (3–8)	2 (1–4)	-2 (-3–-1)	4 (2–5)	4 (3–5.3)	0 (0–0)	0.057	**<0.001**
Pain 3 (score)	4 (2–6)	2 (0–3)	-2 (-2.5–-1)	4 (2–5)	3 (2–5)	0 (-0.3–0)	0.39	**<0.001**
Pain 4 (score)	2 (1.5–5.5)	1 (0–3)	-1 (-2–0)	3 (1.8–5)	3 (1–5)	0 (0–0)	0.81	**<0.001**
*Hemodynamic variables*								
SBP 1 (mmHg)	120 (110–143)	116 (110–144)	-3 (-9–3)	122 (111–142)	123 (110–141)	0 (-7–1)	0.84	0.71
SBP 2 (mmHg)	123 (110–139)	122 (113–139)	0 (-5–7)	124 (109–138)	122 (105–138)	0 (-6–2)	0.84	0.25
SBP 3 (mmHg)	130 (110–138)	120 (110–137)	0 (-10–3)	120 (110–130)	120 (110–129)	0 (-5–2)	0.46	0.55
SBP 4 (mmHg)	125 (115–140)	120 (120–139)	0 (-6–5)	120 (110–131)	120 (110–130)	0 (-10–5)	0.18	0.46
DBP 1 (mmHg)	71 (63–83)	66 (62–80)	-1 (-5–3)	77 (63–85)	74 (62–85)	0 (-4–1)	0.75	0.69
DBP 2 (mmHg)	70 (64–80)	70 (67–80)	0 (-5–5)	71 (64–80)	70 (63–80)	0 (-1–0)	0.34	0.44
DBP 3 (mmHg)	71 (63–80)	70 (66–80)	0 (-4–5)	70 (62–80)	71 (67–80)	0 (-2–6)	0.52	0.64
DBP 4 (mmHg)	80 (70–85)	80 (70–90)	0 (0–7)	80 (65–85)	80 (64–86)	0 (0–6)	0.99	0.83
HR 1 (beats/min)	85 (75–90)	86 (93–91)	1 (-4–4)	85 (72–96)	85 (76–96)	2 (-1–5)	0.75	0.34
HR 2 (beats/min)	85 (80–90)	82 (77–94)	-1 (-6–4)	87 (82–98)	89 (76–99)	1 (-4–2)	0.34	0.91
HR 3 (beats/min)	87 (79–95)	85 (81–98)	2 (-2–5)	88 (80–97)	90 (91–100)	1 (-2–3)	0.52	0.25
HR 4 (beats/min)	85 (80–96)	90 (97–97)	1 (-3–5)	91 (80–96)	91 (81–98)	2 (-2–3)	0.99	0.73
*f*_R_ 1 (breaths/min)	18 (16-22)	19 (16-21)	0 (-2–2)	19 (16-22)	19 (16-24)	1 (-2–3)	0.76	0.42
*f*_R_ 2 (breaths/min)	19 (16-23)	19 (15-21)	0 (-3–1)	18 (16-20)	18 (16-21)	1 (-1–2)	0.52	0.14
*f*_R_ 3 (breaths/min)	18 (16-20)	18 (16-20)	0 (-2–2)	18 (16-20)	20 (17-23)	2 (-1–2)	0.68	**0.005**
*f*_R_ 4 (breaths/min)	18 (16-20)	18 (16-20)	0 (-2–2)	18 (16-20)	19 (18-21)	1 (-1–2)	0.67	0.26
SpO_2_ 1 (%)	94 (92–96)	93 (92–96)	0 (-1–1)	94 (93–95)	95 (93–96)	1 (0–1)	0.30	0.60
SpO_2_ 2 (%)	94 (93–95)	94 (92–96)	0 (-1–1)	94 (93–96)	95 (93–95)	1 (-1–1)	0.41	0.93
SpO_2_ 3 (%)	94 (93–95)	95 (93–6)	1 (0–2)	95 (93–96)	95 (93–96)	0 (-1–1)	0.67	0.15
SpO_2_ 4 (%)	95 (93–97)	95 (93–97)	1 (-1–2)	95 (93–96)	95 (94–96)	0 (-1–2)	0.71	0.56

The results are expressed as the median (interquartile range); SBP: systolic blood pressure; DBP: diastolic blood pressure; HR: heart rate; f_R_: respiratory frequency; SpO_2_: peripheral oxygen saturation. Numbers 1, 2, 3, and 4 refer to the pre- and postapplication moments of the 4 TENS/placebo sessions. ^∗^Comparison between the 2 groups in relation to pre-TENS measurements. ^#^Comparison between the 2 groups in relation to absolute delta values (post-TENS minus pre-TENS measurements).

## Data Availability

The data used to support the findings of this study are available from the corresponding author upon reasonable request.
